# The Endurance of Uncertainty: Antisociality and Ontological Anarchy in British Psychiatry, 1950–2010

**DOI:** 10.1017/S0269889713000410

**Published:** 2014-03

**Authors:** Martyn Pickersgill

**Affiliations:** University of Edinburgh E-mail: martyn.pickersgill@ed.ac.uk

## Abstract

Research into the biological markers of pathology has long been a feature of British psychiatry. Such somatic indicators and associated features of mental disorder often intertwine with discourse on psychological and behavioral correlates and causes of mental ill-health. Disorders of sociality – particularly psychopathy and antisocial personality disorder – are important instances where the search for markers of pathology has a long history; research in this area has played an important role in shaping how mental health professionals understand the conditions. Here, I characterize the multiplicity of psychiatric praxis that has sought to define the mark of antisociality as a form of “ontological anarchy.” I regard this as an essential feature of the search for biological and other markers of an unstable referent, positing that uncertainties endure – in part – precisely because of attempts to build consensus regarding the ontology of antisociality through biomedical means. Such an account is suggestive of the co-production of biomarkers, mental disorder, and psychiatric institutions.

## Introduction

In Britain, antisociality has long been regarded as an area of potential psychiatric concern.^1^ As such, considerable discussion has revolved around its epistemological and ontological status: how should this pathology be studied? What causes it? Whilst hotly debated, these key questions have proved difficult to resolve. Nevertheless, in spite of this characteristic uncertainty, individual mental health professionals continue to have specific ideas about what might cause disorders of sociality – such as psychopathic personality disorder (psychopathy) and antisocial personality disorder (ASPD) – advancing these in contributions to journals and assuming them within their research and practice.

In this paper, I analyze some of the debates pertaining to antisociality as they have played out within British psychiatric discourse. In particular, I am concerned with the place, role, and impact of biological correlates of psychopathy and ASPD.^2^ Twenty-first-century attention to the role of the biological within mental illness continues a long-tradition within British psychiatry (Moncrieff and Crawford 2001); research into “biomarkers” – biological correlates of pathology that have implications for diagnosis, prognosis, treatment and drug development – is not new. Somatic motifs pattern the narratives of psychiatric discourse, developed through and further developing theory, research, and clinical practice.

Yet, so too do other themes. Indeed, the on-going articulation of biological, psychological, and sociological discourse within the mental health professions complicates attempts to rigidly characterize particular research programs or clinical frameworks as, for instance, “biological” psychiatry. Often, we may see the intertwining of concepts and practices. This renders problematic attempts to understand the social life of biomarkers without also attending to (what mental health professionals call) the “biopsychosocial” spaces (see Pilgrim 2002) within which they find mobility, gain traction, and develop salience. Accordingly, in this paper I do not solely analyze discussions of the somatic aspects of disorders associated with antisociality, but rather situate these within a broader examination of the shifting aetiological emphases of psychiatry.

Methodologically, the analysis presented develops from a close reading of research papers, editorials, correspondence, and book reviews pertaining to antisociality within the *British Journal of Psychiatry* (hereafter, BJP) from 1950–2010 (and other key articles). The BJP has long been and remains one of the key Anglophonic journals of psychiatry, and an interrogation of its contents provides an important window into the changing professional dialogues, debates and disagreements that have played out within UK mental health theory, research and practice.^3^ Attention to ASPD and psychopathy is especially warranted given the relative dearth of historical and sociological literature on these categories; this is all the more striking when the profound effects such labels may have on men and women are borne in mind. Whilst the paper focuses on pathological antisociality, however, I suggest that the wider dynamics characterized herein might likewise be apparent for other contested categories, such as anorexia (Gremillion 2003), autism (Silverman 2012), depression (Ehrenberg 2010), and schizophrenia (Metzl 2010).

Investigating the shifting understandings of psychopathy, ASPD, and their ascribed psychic and somatic correlates and causes contributes to a more contextualized appreciation of the place and role of biomarkers within mental health. My account highlights the continuities of current research foci on biomarkers within older trajectories, whilst also admitting the distinctiveness of twenty-first-century approaches. Any novelty, however, is itself a function of the heterogeneity that has so long been evident within psychiatry, enabling individual clinicians and researchers to take as their focus different elements constitutive of the biopsychosocial matrix (and, in the process, further disunifying the profession).

## The Social Life of Biomarkers and the Anarchy of Science

The potential biological correlates, and perhaps causes, of psychopathy and ASPD have structured debates about these conditions in key ways. Indeed, biomarkers have come to play an especially important role within medicine and the life sciences in general. Used in screening for disease, as targets in pharmaceutical research, and as indicators of exposure to environmental hazards, the idiom of biomarkers can be found across the discursive terrain of science and medicine internationally (Metzler 2010).

A variety of scholars from the social sciences have advanced critical commentary on the ascendance of biomarkers; as Waldby argues, their use to configure individual disease risk “simultaneously expand[s] the potential market for therapeutics while clearly delineating the geographical and demographic borders of that market” (Waldby 2009, 262). More generally, the use of biomarkers constitutes a variety of “ethical tension zones” (Caux et al. 2007). In psychiatry, there may be particular issues associated with the labelling of individuals as “risky,” which could contribute to the further stigmatization of and use of pharmaceuticals by children and adolescents (Singh and Rose 2009).

Of course, not everyone shares these concerns. As well as holding promissory value for researchers in the life and medical sciences, social scientists too have called for more work that brings together measurements of biomarkers with sociological analysis to produce what Timmermans and Haas (2008) refer to as a “sociology of disease.” Yet, despite the ways in which biomarkers, like other diagnostic “technologies,” have come to be seen by many as a means of offering “a more ‘objective’ approach to diagnosis than reliance on the vagaries of symptom reporting” (Armstrong et al. 2007, 581), their use may in fact further complicate the multifaceted nature of illness. As anthropologist Margaret Lock and colleagues (2006) discuss for the case of Alzheimer's disease, research findings about biomarkers “create uncertainties and destabilization” (Lock et al. 2006, 285), raising profound questions about the ontology of disorder; if pathological indicators may even be present when an individual seems healthy, what, then, is the disease?

Such questions are associated with a long and distinguished philosophical tradition (Canguilhem 1991), and it is not my intention to either summarize or answer them here. Eschewing a normative response to ambiguity within medicine, I instead take a descriptive approach, examining the ways in which disorders of antisociality have been constituted and located within psychiatric theory, research, and practice over the last sixty years. In so doing, I draw in part on the epistemology of Paul Feyerabend (2010, 2011). In spite of the normativity of Feyerabend's work, and its focus on ways of knowing, rather than ways of standardizing or defining ontologies, it holds insights for the sociological history I present here. Feyerabend's philosophy is itself historical and empirically grounded; his epistemological positions and proscriptions are based on a careful analysis of the development of the natural sciences. Of particular interest here is his thesis that rationalism is an insufficient theory of knowledge production and circulation. Rather, Feyerabend argues that anarchism has been and needs to be an essential aspect of science; a view he advances with force and feeling:

*Unanimity of opinion may be fitting for a rigid church, for the frightened or greedy victims of some (ancient, or modern) myth, or for the weak and willing followers of some tyrant. Variety of opinion is necessary for objective knowledge. And a method that encourages variety is also the only method that is compatible with a humanitarian outlook*. (Feyerabend 2010, 25, emphasis in original)

These are strong words indeed, and – to be clear – it is not my aim here to suggest that they should necessarily be heeded by psychiatrists or anyone else. However, Feyerabend's thesis of methodological anarchism and the inherent disunity of science – which has also been addressed by philosophers such as John Dupré (1983) – can also be read as a descriptive statement of how science evolves. It is this empirical claim that I wish to develop. For psychiatry, a kind of *ontological anarchy* is apparent (at least, for the specific case I examine herein). Through this concept, I intend to capture the diverse theories and practices that have attempted to clarify the murky pathological image that has so long been characteristic of antisociality. Indeed, I suggest that it is at least in part precisely through this diversity that a stable referent for terms such as “psychopathy” or “antisocial personality disorder” has so far escaped the sophisticated attempts of psychiatrists to constitute it. In other words: the variety of theories and experiments associated with pathological antisociality creates a range of parallel trajectories along which research might progress, increasing the breadth of perspectives available to mental health professionals from which to articulate the ontology of conditions like ASPD and psychopathy. This, perhaps paradoxically, makes it more difficult for a working consensus on the nature of these disorders to emerge and from which widely-agreed upon claims about development, identification and treatment can be made. As a consequence, further research and conceptual work is propelled. Ontological anarchy is thus – to a degree – autopoietic: it is a response to the uncertainties inherent to dealing with antisociality in a psychiatric context, as well as an engine powering the generation of yet more ambiguity.

In what follows, I chart some of the uncertainties associated with psychopathy from the origins of the concept, before moving to examine a range of biological understandings of antisociality debated in mid-twentieth-century Britain.^4^ I then discuss how these sat alongside more psychosocial conceptions, and the ways that they were often integrated. Moving forward, into the late twentieth century, I document the great extent to which much earlier uncertainties remained, and the hopes of new technologies of standardization to clarify these ambiguities. In the new millennium, however, doubts and frustrations continue to be apparent, and, as we will see, even the promissory practices of neuroscience and molecular genetics have, so far, failed to provide consensus on the ontology of antisociality.

## British Psychopaths

Since at least the 1950s, psychopathy in the UK has been recognized as a severe personality disorder characterized in individuals who have wantonly and repeatedly committed severely antisocial acts (see Berrios 1996; Werlinder 1978). Historian Henry Werlinder (1978) has largely attributed this conception to the influence of David K. Henderson on UK psychiatric thought in the second quarter of the twentieth century (see also, Fox 1964; Saß and Herpertz 1995; Saß and Felthous 2008; Shorter 2005). Henderson, a leading Edinburgh psychiatrist and author of *Psychopathic States* (1939), was influenced by German and American psychiatric traditions, both of which were evident in his highly influential *Text-book of Psychiatry*, co-authored with Robert D. Gillespie and first published in 1927 (Gelder 1991; Hilton 2005; Werlinder 1978). Henderson took psychopathy to be a constitutional inferiority of personality, expressed through criminal behavior (Gelder 1991; Werlinder 1978). To assert that psychopathy was constitutional was not, however, to say that it was necessarily an inherited condition, or even biologically determined: the notion of constitutional factors emphasized biology, certainly, but also a contingent interaction between biology and environment (Berrios 1996; Rosenberg 2007; Werlinder 1978).

Werlinder (1978) has argued that Henderson's psychopathy was an ambiguous construct: exactly what caused the condition was unclear. Time did little to clarify matters; in the 1950s, when a new Mental Health Act for England and Wales was being developed, psychopathy remained conceptually vague – as commentators like leading social scientist and magistrate Barbara Wootton (1959) were acutely aware. Critical of the use of the term psychopathy, Wootton wryly observed that one definition of the disorder given by the Royal Medico-Psychological Association (the forerunner of the Royal College of Psychiatrists) essentially reduced to the following statement: “psychopaths are extremely selfish persons and nobody knows what makes them so” (Wootton 1959, 249). Resonating with US debates about psychopathy (Pickersgill 2010), Wootton's doubts about the legitimacy of the concept were closely related to its ambiguity. As she noted: “The volume of the literature on the subject of psychopaths is rivaled only by the depth of confusion in which this literature is steeped” (Wootton 1959, 250).

Prominent clinicians remarked similarly. Aubrey Lewis, widely regarded as one of the most important figures in twentieth-century psychiatry, observed: “Every text-book of psychiatry discusses this abnormality, but almost always ambiguously because the authors do not make clear why it should be regarded as an illness” (Lewis 1953, 119). This was a direct consequence of the dominant means of recognizing psychopathy; essentially, persistent antisocial conduct. This raised a particular problem: that of the individual who is “sociopathic without being psychopathic” (ibid., 116) – a dilemma especially pertinent in medico-legal discussions, within which the ambiguous “quantitative abnormality of impulse” that could be associated with psychopathy was regularly enrolled and “so often urged as a medical explanation for lessened responsibility in crimes of violence” (ibid., 119). Accordingly, “it would seem, then, that until the category is further defined and shown to be characterized by specified abnormality of psychological functions, it will not be possible to consider those who fall within it to be unhealthy, however deviant their social behavior” (ibid.).

As long as the concept of psychopathy has had a place within British psychiatry, then, it has been characterized by uncertainty and confusion. Lewis’ own solution, like those of many psychiatrists over the last 60 years, was to seek to destabilize the diagnostic category through the reflexive production of uncertainty regarding the ontology of its referent, only to appeal to new knowledge practices in order to promote re-reification in the future (cf. Moreira et al. 2009). In essence, only by rendering problematic the taken-for-granted could psychiatric innovation and clinical justification be assured.

## The Mark of the Psychopath

In the 1950s, British psychiatry was, in terms of the conceptualization and treatment of mental disorder, a “broad church” (Ingleby 1998, 300). Nevertheless, many psychiatrists tended to emphasize its somatic aspects (Micale 2000), and pharmacological treatments such as chlorpromazine were widely used and associated with shifts in patient care away from the asylums.^5^ Psychopathy (and personality more generally) was not exempt from being understood through a biological framework (Moncrieff and Crawford 2001; Parker et al. 1995), and aspects of an individual's soma, including body size and physique, were thought to vary with antisociality and aggressiveness (e.g., Gibbens 1957; Rees 1950a, b, c). The U.S. psychologist William H. Sheldon, whose work focused on “somatotyping” (i.e., linking body-type to personality), was especially influential (Rafter 2007).

In particular, high-profile researchers such as Hans J. Eysenck highlighted and popularized the importance of heredity in determining an individual's personality (Eysenck and Prell 1951). Eysenck was a psychologist at the London-based Institute of Psychiatry (IoP), a prestigious center of international excellence in psychiatric research, home to a number of opinion-leaders. A prominent critic of psychoanalysis, Eysenck judged this tradition to have a poor scientific basis (Baruch and Treacher 1978; e.g., Eysenck 1953). His views on personality and its pathologies thus stood in stark contrast to the psychoanalytic model(s) through which many U.S. psychiatrists understood the development of mental disorder (Sclare 1953).^6^

The influence of Eysenck's personality psychology was felt strongly within mid-twentieth-century British psychiatry and psychology (Rafter 2006), and it informed much research into psychopathy. He contributed significantly to the quantitative study of personality using statistical techniques (Buchanan 2010; Crown 1983); to Eysenck, personality was calculable, knowable through tools such as his Maudsley Personality Inventory (a commonly used means of scoring personality traits).^7^ As we will see, such technologies helped to foster the conditions of possibility for the creation and employment of late-twentieth-century personality inventories such as the Hare Psychopathy Checklist.

Eysenck was widely cited by articles in the *Journal of Mental Science* (JMS; forerunner of the *British Journal of Psychiatry*), and somatic ideas about psychopathy permeated this periodical. Contributor Alexander Kennedy, for instance, considered psychopathic personality disorder to be “usually” constitutional (Kennedy 1954, 873), “inborn or due to failure of post-natal development or maturation” (ibid., 876). Kennedy thought that “it is possibly by means of comparatively small structural damage, or by interfering at critical phases of development, to produce an individual who *lacks the physical means of being morally aware*” (ibid., 879; emphasis in original). Underscoring the ambiguity of the category, Kennedy's psychopaths were a heterogeneous group. Under the rubric of psychopathy came “moral-oligophrenics, antisocial perverts, anarchists, vengeful paranoids, the angry and explosively violent who constantly offend against the law” (ibid., 878). These diverse individuals seemed united only by their acute and essential antisociality. Kennedy thought that psychiatrists might “tame” a beastly psychopath through environmental manipulation, but his “savage heart” would be ultimately unaffected (ibid., 873). His account was thus evocative of Henderson's inter-war constitutional formulation of psychopathy. Not seemingly genetically determinist, Kennedy nevertheless considered that it would be a considerable challenge getting a psychopathic “leopard” to “change his spots” (ibid.).

In line with these essentialist ideas, explicitly cerebral understandings of psychopathy were also apparent in mid-twentieth-century psychiatric discourse. For instance, electroencephalography (EEG), a popular tool for the measurement of the electrical activity of the brain and routine diagnostic aid for neurological conditions such as epilepsy (Borck 2008), was investigated as a means of enhancing the reliability of, and decreasing the uncertainty associated with, the diagnosis of psychopathy (Werlinder 1978).^8^ The hopes and expectations regarding the clinical utility of EEG resonate with more recent anticipatory claims regarding late-twentieth-century neurotechnologies (Borck 2008). The ontological imaginaries embedded within the practices of electroencephalography were instantiated in the clinic by the taking of EEG recordings from individuals labeled as psychopathic. These results were used to show how the pattern of brain electrical activity was different between psychopathic and non-psychopathic subjects, implying a cerebral abnormality in the former (D. Hill 1956; Gibbens et al. 1959).

The common use of EEG underscores the confidence of many psychiatrists that psychopathy was a biological disorder with a clearly defined signature in the brain. Indeed, some viewed leucotomy (a surgical operation on the brain) as an effective means of reducing aggressive behavior in psychopaths (Thorpe and Hardman 1952). However, these endeavors attracted some debate. For instance, on 9 October 1951, Desmond Curran, in his Presidential Address to the Section of Psychiatry at The Royal Society of Medicine, voiced concern that abnormal EEG patterns could be used to give leniency to criminals with no sign of mental disorder (Curran 1952). Again, such discussions foreshadow twenty-first-century attempts to employ neuroimaging tools to add empirical flesh to the bones of philosophical discussions about responsibility (Pickersgill 2011a).

Nonetheless, in spite of the efforts of various investigators, scientific research around psychopathy undertaken in the 1950s and 60s was not found to translate easily into clinically useful knowledge – EEG readings did not map easily onto nosological entities (Craft et al. 1962, 581). Indeed, research detailing disappointing results about the lack of diagnostic value of EEG could be seen more generally in psychiatry at this time (e.g., Turton 1958). If EEG was, in theory, a sensible means of rendering the ontology of mental disorder more transparent, in practice its application had the effect of making psychopathology yet more unclear: “The empirical approach to EEG data has produced, not increasing simplification, but increasing complexity and even certain strong points of clinical application have had to be abandoned” (D. Hill 1956, 265).

The cerebral conceptions of psychopathy that the use of EEG implies at once recall nineteenth-century ideas about the neurologic origins of mental disorder (Rosenberg 2007), whilst also setting the scene for more recent attempts to identify cerebral markers of antisociality (Pickersgill 2009). As we will see, the obfuscation of an already ambiguous construct that was performed through the use of this technology likewise has important parallels with later research.

## Disarranging Ontologies

In spite of the somatic slant of much discourse and practice regarding psychopathy in Britain in the 1950s and 60s, some psychiatrists nevertheless recognized a role for the environment in the development of this disorder. As one commentator argued in the JMS: “The grossest psychopaths appear where heredity and environment have conspired jointly to produce deprivation or arrest of the moral and emotional development, a chronic emotional malnutrition as it were over years” (Sands 1953, 124). Clearly, whilst biological accounts of antisociality were common, other ways of thinking about the origins of psychopathy were possible.

That developmental narratives drawing upon environmental and psychic themes were considered legitimate to advance was in no small part due to the long tradition of “social psychiatry.” Although the dominant theoretical underpinnings and clinical practices associated with this tradition have shifted over time (Neve 2004), the various formulations of social psychiatry all imply a particular ontology of mental illness that emphasizes the important role of “the environment” in the development and expression of psychiatric disorder.^9^ In one clinician's words, “mental illness is to some extent a disease of civilization” (E. Hare 1952, 579). Accordingly, “a programme of social psychiatry – the prevention and treatment of mental disease by social measures – must be based” on statistical research into two key questions: “how much of mental disease is caused by the conditions of civilization, and what particular factors are most responsible for causing it?” (ibid.). Environmental and somatic narratives thus played out together in mid-twentieth-century psychiatric discourse. Indeed, some individuals made active efforts to integrate these discourses; Aubrey Lewis, for instance, was especially inspired by the “psychobiology” of Swiss-born, U.S.-based psychiatrist Adolf Meyer.^10^ Such conceptualizations reflected – and furthered – the theoretical and practical eclecticism that Baruch and Treacher (1978) have argued to be longstanding within British psychiatry.

As indicated above, the heterogeneous understandings of psychiatric disorder in general engendered similarly varied accounts of specific conditions. Thus, the aetiological narratives of psychopathy that appeared within journals like the JMS were wide-ranging (Fox 1964). Factors such as economic and emotional deprivation as a result of absent parents were implicated in the development of personality disorder and antisocial behavior more broadly (e.g. Brown and Epps 1966; Frommer et al. 1972; Koller 1971; Scott 1977; Tennent et al. 1971; Wolkind 1974). These sat alongside more organic conceptions throughout the 1960s and 1970s (Nielson 1971), during which time wider debates were being played out regarding the links between an individual's genetics (particularly, the XYY genotype) and aggressiveness.^11^ Correlations between physique and personality type continued to be explored (Borgaonkar et al 1972; Nielson and Tsuboi 1970), as were longstanding associations between violence and epilepsy (see Gunn 1974; Gunn and Bonn 1971; Hills 1971; Kristianson 1974).^12^

Like the integrationist psychiatry of key figures like Aubrey Lewis, some clinicians and investigators at the coalface of practice and research acted as what might be called “ontological bricoleurs,” molding together different developmental accounts into a complex conceptual framework. For instance, Michael Craft, a consultant psychiatrist at Balderton Hospital in Nottinghamshire, published articles both on EEG analysis of individuals detained within a “Psychopathic Unit” (Craft et al. 1962), and on the relationship between childhood adversity and personality disorder (Craft et al. 1964). In the latter case, he argued that the psychological and social structural effects of parental absence contributed to the development of psychopathology. The discourse and practice of British mental health professionals complicated historiographical distinctions between “biological” and “psychological” styles of psychiatry (cf. Pickersgill 2010, Pickersgill 2011b; Sadowsky 2005).^13^

In the mid-twentieth century, aetiological narratives of both psychopathy and antisociality generally were nothing if not diverse. They ranged widely between the ontological realms of psyche, soma and society and the various topologies constitutive of them (e.g., parental absence, dominant mothers; genetics, neurology; childhood abuse, economic deprivation). As such, there was no stable consensus regarding the development of psychopathy; no specific markers – be they biological or otherwise – could be concretely pinned down and agreed upon. Rather, to paraphrase Feyerabend, in the aetiological discourses of psychiatrists, it was almost a case of “anything goes” (Feyerabend 2010, 8) – at least, within the broad toolkit of pathological determinants generally available to British psychiatrists to build legitimate models of psychopathy.

## In Search of Standards

Disease classifications and diagnostic standards are central features of contemporary biomedicine, ordering knowledge production and helping to delimit uncertainty (Bowker and Star 1999). Alongside the ambiguity regarding the causes of psychopathy were key questions relating to how the disorder should be diagnosed. Answers were not readily forthcoming: since there was “no generally agreed system of classification” (Pritchard and Graham 1966, 605), diagnostic practices varied “greatly from one doctor to another” (ibid.).^14^ Particularly problematic was the tendency of psychiatrists to classify antisocial personality most commonly in individuals with “a history of repeated criminal offences or violent and aggressive behaviour” (ibid., 606). This practice made it harder to establish firm boundaries between individuals with a psychiatric disorder, and those who were “merely” extremely antisocial. There was agreement at least that psychopathy was socially, as well as clinically, problematic: “a community problem, not only to themselves but to their family doctors, social workers, and others who become involved in their affairs and treatment” (Craft 1968, 813). However, a consensus regarding diagnostic practice was far from imminent.

Little had changed by the 1970s; as one contributor to the BJP noted:

Although the term ‘psychopath’ continues to be used widely in clinical practice and research, doubts are frequently expressed as to whether the concept has a substantive referent. As the history of the concept indicates, it has gradually come to be used to identify a class of antisocial individuals by reference to psychological abnormality, but difficulties have arisen since the necessary and sufficient conditions of membership in the class have not been made explicit. (Blackburn 1975, 456)

Psychopathy was still commonly considered, as famously stated by Sir Aubrey Lewis, a “most elusive category” (Lewis 1974). However, this was not just a problem for psychiatrists and other health professionals. Psychopathy also occupied an important position within mental health law, and therefore presented a challenge for policymakers and lawyers. Indeed, a key 1975 report on mentally disordered offenders (the Butler Report) recommended the category be abandoned altogether and be replaced with the broader and somewhat less controversial term, “personality disorder” (McCallum 2001; Wootton 1980). Nevertheless, the use of the psychopathy construct persisted; as criminologist Philip Bean complained, “Years of [clinical and legal] deliberation about the psychopathic patient appear to have led nowhere” (Bean 1979, 102).

The “perennial problem” (ibid.) of psychopathy endured into the 1980s, and mental health practitioners and researchers continued to find its ambiguity troubling (Grounds 1987). In 1981, psychologists William Davies and Philip Feldman noted that “while there are many signs used to justify such a label there is no clear agreement on which, if any, are crucial ones” (W. Davies and Feldman 1981, 329). Accordingly, the validity of the psychopathy concept was “increasingly questioned, and its use little examined, in spite of the fact that when a person is diagnosed as a psychopath by a forensic specialist it can have very severe consequences for him” (ibid.). Davies and Feldman conducted a study examining how psychiatrists used the term psychopathy in practice. They found that though it was commonly deemed useful, clinicians “recognized a large number of diagnostic signs, though with much disagreement about the exact importance of each” (ibid.). Thus, if the ontology of psychopathy remained uncertain, the means of identifying individuals with the disorder was perhaps even more so.

## Towards Standardization

In the 1980s, significant changes were taking place within British psychiatry, bringing with them a new climate of diagnostic certitude. As with the U.S., institutional shifts were made towards a more openly “biological” psychiatry (Busfield 1998; Pilgrim 2002). Such a somatic emphasis within the psychiatric imaginary matched the biomedical framework of the third editions of the American Psychiatric Association (APA) Diagnostic and Statistical Manual of Mental Disorders (DSM-III), released in 1980, which facilitated the uptake of the nosology throughout the world.^15^ As historian and psychiatrist David Healy (2002) has shown, the endorsement of the DSM by pharmaceutical companies contributed to the speedy translation of U.S. frameworks for diagnosis into British discourse, and it did not take unduly long for the text to be used in the training of U.K. psychiatrists (Macaskill et al. 1991). At the same time as the DSM-III was released, debates were being held regarding what would become of the 1983 Mental Health Act of England and Wales. With its precise descriptions of psychiatric disorder, the DSM-III was, it seems, alluring to U.K. clinicians in search of specific and legitimate standards for contested mental disorders.

One such “American” diagnostic entity was “antisocial personality disorder” (ASPD).^16^ A clinically-useful shorthand for the pathological antisociality that the psychopathy construct had long been used to try and capture, ASPD proved appealing to many psychiatrists. In marked contrast to pychopathy, explicit criteria and guidelines for the recognition and diagnosis of ASPD were set out within the DSM, and the subsequent development of the Structured Clinical Interview for DSM-III personality Disorders (SCID-II) helped to make the disorder “visible.”^17^ ASPD could therefore be standardized, ensuring that the DSM-III was the “right tool for the job” (Clarke and Fujimura 1992) of generating stable biomedical knowledge regarding antisociality. “Modern medicine,” historian Ilana Löwy has pointed out, is “a trans-national endeavour” (Löwy 2007, 466); with ASPD, British clinicians and investigators could also more firmly ensconce themselves within international research and debate on personality disorder.

The DSM did not stand alone, however. Alongside the new category of ASPD was the World Health Organization's 1978 International Classification of Diseases (ICD-9) in 1978, which contained a category of “dissocial personality disorder,” similar to ASPD. However, as noted earlier, the ICD appeared to have less traction within UK forensic research than the DSM-III.^18^ More importantly still was a novel means of standardizing psychopathy. In 1980, Canadian psychologist Robert Hare released the first version of his influential Psychopathy Checklist (PCL): a tool for the characterization of psychopathy, based on the concept of the disorder advanced by Hervey Cleckley in mid-twentieth century North America (R. Hare 1980). Hare's Checklist became important for research internationally, including (neuro)biological studies. The 1991 Revision (PCL-R) is today commonly regarded as the “gold standard” for scientific investigations into the disorder. Both Hare's and the APA's standards came to have significant traction within U.K. research, feeding into clinical discourse and leading to fresh debate regarding how these categories relate to each other – and, thus, how the ontology of pathological antisociality can best be bounded (Pickersgill 2011c).^19^

Despite the uptake of the DSM personality disorders within the literature, some professionals – including various influential figures – contested the criteria and the ontological assumptions underpinning them. Psychiatrists influenced by theoretical traditions within personality psychology, for instance, recoiled against the categorical diagnostic within the DSM. These psychiatrists thought personality disorders lay “at the extreme of a multidimensional continuum” (Tyrer and Alexander 1979, 166) of (statistically) normal personality; the notion that there were discrete personality disorder types that individuals either were or were not was rejected. Others, such as IoP faculty members Glyn Lewis and Louis Appleby, argued that DSM definitions of personality disorders were vague, making them unreliable diagnostic categories. Lewis and Appleby even proposed that “the concept be abandoned” (Lewis and Appleby 1988, 45).

However, whilst many held strong views on personality disorders, few appeared to feel that these diagnostics should be completely omitted from the psychiatric vernacular. As one response to Lewis and Appleby argued, “Only when it has been shown that the concept of personality disorder holds no validity should it be discarded” (Cook 1988, 701). Indeed, it is precisely because many viewed personality disorder diagnostics to be administratively necessary and “clinically important” (Rutter 1987, 454) that such debate raged.

Thus, the DSM personality disorders might have been standards, but they were not, many felt, necessarily very good ones. ASPD was particularly contested. A “questionable diagnostic entity” (ibid., 452), it cast into sharp relief the tensions mental health professionals had long faced when adjudicating between normality and pathology. Of particular concern was the wide-range of individuals who might legitimately be diagnosed with ASPD: “both transitory delinquents and continuous antisocials” (Stone 1993, 307) stood under the umbrella of this diagnostic category.^20^ ASPD could thus apply to the merely criminal as well as the truly personality disordered, pathologizing “normal” social deviancy.

Such disquiet had long been associated with attempts to formulate antisociality within a medical rubric; as we have seen, Barbara Wootton (1959) highlighted the problematic circular logic of psychopathy (i.e., extreme antisocial acts may be taken to be indicative of mental disorder, an assertion which is itself then validated by pointing to the unusually antisocial behavior of the individual labeled psychopathic).^21^ The endurance of these concerns suggests the degree to which some psychiatrists were reflexive about their conceptualizations of pathology, whilst also underscoring how regularly and easily “philosophical” concerns about personality disorders associated with antisocial behavior were side-lined. Such “practical uncertainty work” (Pickersgill 2011c) was essential in enabling clinical and scientific professionals to go about the job of managing patients and undertaking research without being paralyzed by fundamental ontological questions.

If ASPD attracted similar kinds of critique as psychopathy had in the past, it was not, however, because psychiatrists necessarily took these categories to be “the same.” Rather, the relationship between ASPD and psychopathy was problematic. Though both classified antisociality, the degree to which they could be said to capture the same psychopathology was not self-evident. On the one hand, some considered ASPD and psychopathy to be markedly different entities. Psychopathy was viewed as a severe form of personality disorder associated with traits such as lack of empathy, whereas ASPD applied to individuals who were indisputably delinquent, but lacked the severe antisociality characteristic of psychopathy. On the other hand, as psychologist Ronald Blackburn (1988) argued, in practice, ASPD and psychopathy could be taken to be overlapping entities (see also, more recently, Cawthra and Gibb 1998).

Such uncertainties were not solely clinical concerns; they were also construed as a potential impediment to the advancement of scientific knowledge concerning personality disorder. Though at least some investigators (e.g., Virkunnen 1983) considered that the increased “accuracy” of the DSM diagnostic criteria for standardizing personality disorder had paved the way for better, more scientific studies, others thought the use of similar behavioral proxies (such as criminality) for both psychopathy and ASPD was concerning (see, for instance, Dolan 1994).

Boundaries between psychopathy and ASPD were thus not easily erected. Since a consensus about the nature of psychopathy had always escaped British psychiatry, definite boundaries were difficult, if not impossible, to secure. This in turn blurred the edges of ASPD, which the authors of the DSM had invested considerable energy in sharpening as much as possible (Pickersgill 2013a). That boundaries were deemed necessary to police at all, however, is telling: though ASPD (or even dissocial personality disorder) might simply have replaced psychopathy within the psychiatric idiom, the coexistence of these concepts draws our attention to the significant traction of the latter category in spite of its uncertain ontology.

## Structuring Aetiology

A key set of developments within biomedicine in the last century was the proliferation and increased sophistication of molecular genetics techniques (Keller 2000). In psychiatry, such approaches were popularized by influential figures like psychologist Robert Plomin (Plomin et al. 1980; Plomin 1986; Plomin et al. 1988; Plomin and McClearn 1993; Plomin 1994), a professor of behavioral genetics at the IoP since 1994. Genetic motifs were evident in discourse on personality disorder; some British psychiatrists advanced genetic ideas and research in journals like the BJP (McGuffin and Thapar 1992), as did clinicians and researchers from abroad (Virkkunen 1983). Indeed, genetic perspectives on antisocial behavior and violence broadly seemed increasingly visible in the late twentieth century. In particular, the research of psychologist Avshalom Caspi (Professor of Personality Development, also at the IoP) investigating gene-environment interactions in violent individuals came to be especially influential (e.g. Caspi et al. 2002).^22^ Genetic science was clearly an important ontological anchor for various kinds of interests in antisociality.

Alongside their interests in genetics, British psychiatrists – like their colleagues in the U.S. – were also turning ever more towards the neurosciences. The use of brain imaging technologies such as Positron Emission Tomography (PET) and Magnetic Resonance Imaging (MRI) helped to constitute new forms of objectivity (Beaulieu 2001, Beaulieu 2002), and neuroimages had a broad valence in scientific, clinical and popular cultures (Dumit 2004; Joyce 2008). Psychiatrists increasingly drew on neurobiological explanations for the aetiology and expression of mental disorder, and interventions that acted directly on the brain through neurotransmitters were widely accepted and used (Healy 2004; Micale 2000; Rose 2007).

Neuroscience was construed as a powerful way through which the ontology of psychopathology could be rendered transparent; as an editorial in a BJP supplement entitled “Brain imaging in psychiatry” concluded:

The application of neuroimaging techniques in the study of psychiatric disorders has provided one of the most dramatic developments in biological psychiatry. There is little doubt that integrated, structural-functional techniques will lead to major advances in the understanding of the biology of psychiatric disorders, refining their classification and guiding management. (Abou-Saleh 1990, s9)

The brain was thus constructed as what Brown and Kraft (2006) might call “promissory matter,” a bodily substance around which numerous anticipatory claims revolved. Key amongst these was that knowledge of the brain would improve understanding of psychiatric disorders and, consequently, enhance practice. Nevertheless, this was not a radical shift so much as an amplification of established neurologic hopes in British psychiatry. As explored earlier, EEG technologies had routinely been used in hospitals housing individuals characterized as psychopathic, and the practice continued into the late twentieth century. Psychologist Richard Bentall recalls: “When I worked in a High Security Hospital after qualifying as a clinical psychologist in the mid-1980s, EEGs were routinely taken from patients. The reports made by the electroencephalographer usually made vague reference to ‘possible’ and ‘non-specific’ abnormalities and were almost always useless for clinical purposes” (Bentall 2003, 161).

Papers drawing on EEG methodologies to investigate psychopathy were also still reported in the BJP; for example, Howard and colleagues documented in 1984 that individuals categorized under the Mental Health Act as psychopathic had significantly different EEG results from offenders not recognized as psychopaths (Howard et al. 1984; see also Shagrass et al. 1984).^23^ At the same time, techniques such as PET and MRI began to be increasingly employed by researchers interested in the organic correlates of antisociality. U.S. psychiatric researchers Nora D. Volkow and Lawrence Tancredi (1987), for instance, reported a study in which they used PET to elucidate neural substrates of violent behavior. Although neuro-technologies and the physiological assumptions implicit within them have shifted over time, neurologic constructions of personality disorder have thus been a persistent feature of British discourse. Nevertheless, it seems clear that their use and influence have been gradually extended over the last 20–30 years.

However, just because researchers operated within a neurologic framework did not mean that the disorders they studied were reduced solely to the isolated brain. As Volkow and Tancredi concluded in their paper on the neurobiology of antisociality:

It is important to emphasize that we do not attribute the violent behaviour of these cases solely to brain abnormalities, but rather that the occurrence of the violent outbursts are facilitated by the cerebral dysfunction. Most of the violent behaviour seen in patients probably represents complex interactions between various neural systems, neurotransmitters, hormones, environmental stimuli, and learned responses. (Volkow and Tancredi 1987, 672)

Psychiatrist and clinical researcher Mairead Dolan (1994) also sought to re-introduce a neuroscientific perspective to the understanding of psychopathy; she too considered that though there was “evidence of a genetic influence” (ibid., 152) in the development of the disorder, an individual's genetic profile did not necessarily predetermine pathological antisociality, or that this had an exclusively somatic aetiology: “It is unlikely that either congenital or acquired [neurobiological] deficits alone could account for the development of psychopathy, but they may be interacting with adverse environmental circumstances to predispose the individual to act in an antisocial way” (ibid.). Neuroscientific analyses of constructs such as ASPD and psychopathy therefore granted ontological primacy to biology, but nevertheless positioned “the environment” as playing a part in the development and expression of these disorders (Pickersgill 2009, 2011b; cf. Hedgecoe 2001; Shostak 2003; Rose 2007).

Such psychosocial sentiments on behalf of primarily “biological” researchers must, however, be read within the context of a backlash against “reductionism” that was occurring even as biomedical approaches to theory, research and practice within British psychiatry were in ascendancy. “Biological psychiatry” was alarming to many who favored more “holistic” approaches to understanding and intervening in psychiatric disorder. Psychiatrists averse to neuroscience interpreted neurobiological models of aetiology and psychopharmaceutical therapies as “reductionist,” arguing that by foregrounding biological aspects of aetiology, the importance of psychological and sociological factors would be lessened – an approach that would ultimately be detrimental to therapy. The U.S. psychiatrist Leon Eisenberg, for instance, remarked in his 1986 Eli Lilly Lecture before the Royal College of Psychiatrists:

The peril is that psychiatry may come to focus exclusively on the brain as an organ and to overlook the experience of the patient as a person. We have been for so long pilloried by our medical and surgical colleagues as witch-doctors and woolly-minded thinkers that it is tempting to seek professional respectability by adhering to a reductionistic model of mental disorder. We may trade the one-sidedness of the “brainless” psychiatry of the past for that of a “mindless” psychiatry of the future. (Eisenberg 1986, 500)^24^

In his lecture, Eisenberg cast light on why many psychiatrists found biological approaches alluring (e.g., they imbued “respectability” on practitioners), whilst also illuminating the dangers of reducing patient distress to nothing more than dysfunctional soma. As others have noted, such critique is international in its nature (Beaulieu 2003; Leibing 2008), and as longstanding as its referent (Rosenberg 2007); certainly, evidence of “anti-reductionism” has appeared in a number of forms within the BJP over the last fifty years (e.g. Bannister 1972; Krauss 1972; Lake 1972; Walton 1966). That such sentiments are so readily apparent even within the pages of such an “establishment” journal underscores the sometimes uneasy co-existence of “techno-somatic” psychiatry (Pickersgill 2010) with other styles of thought and practice. “Biological” epistemologies and the ontologies they constituted continued to sit alongside other approaches that emphasized the importance of psyche and society (as also reflected upon by Ingleby 1998; Gittins 1998; Moncrieff and Crawford 2001; Porter 2002; Samson 1995).

With this in mind, it is not surprising that some contributors to the BJP not only gave credence to psychosocial factors in primarily biological articles, but that others concerned themselves specifically with the environment. In 1983, for instance, Mark A. Stewart and C. Susan de Blois from the United States argued that sons learnt antisocial and aggressive behavior from their fathers. More common, however, was an orientation towards some form of biopsychosocial, model, where genetics, environment and psychology were all taken to be salient in the development of pathology (see, for example, Coid 1999; McGuffin and Thapar 1992; Rutter 1987; Tyrer et al. 1991). Yet, as prominent personality disorder commentator Jeremy Coid noted, much work remained to be done in order to more precisely illuminate the aetiology of these complex conditions: “Aetiology may be multifactorial, with personality disorder the outcome of multiple, constitutional and/or environmental factors, which are additive until they overcome a certain threshold, require a sufficient level of severity or most occur at a critical developmental phase during the lifespan” (Coid 1999, 530). An uncertain aetiology might be clarifed though the analysis of developmental risk factors; entities which influenced the likelihood of developing personality disorder, and which were amenable to delineation and examination by investigators (e.g. Coid 1999; Reitsma-Street et al. 1985). A family history of personality disorder was, for instance concluded by Coid (1999) to be a key risk factor. Nonetheless, how these risk factors mapped onto aetiology was not straightforward.

In the late twentieth century, British psychiatry was, it appears, moving further towards a biomedical orientation; as public spending on medical/biological research increased (Micale 2000), a techno-somatic ethic became ever more evident. Of course, it remains an open question as to whether mental health was actually becoming more “biological” in terms of its research and therapeutic emphases, or whether biological discourse was simply more visible and research in this area more expensive than in previous decades. However, as with the U.S., an ontological move had been gradually effected such that scientists, health professionals and publics increasingly seemed to view health and illness through a molecular lens (Kerr 2004; Novas and Rose 2000), engendering an epistemological logic within psychiatry which dictated that psychopathology was best understood through genetic and neuroscientific technologies. Yet, even as biomedical ways of knowing and acting upon patients increased in prestige and prominence, other styles of thought and action that emphasized the role of psyche and society in the development and expression of psychopathology were visible within clinical discourse and practice. Thus, British psychiatry's tradition of theoretical and practical eclecticism (Baruch and Treacher 1978) endured.

For personality disorder this ontological diversity was not without its limitations, for if aetiology was broad it was also unclear. Developmental mechanisms remained “extremely difficult to determine” (Tyrer et al. 1991, 469), and the literature on the subject was regarded by many as “confusing and inconsistent” (Dolan 1994, 157). As the century closed, much work remained to be done to elucidate the precise pathways through which disorders of sociality developed (Coid 1999), and a consensus around the aetiologies of ASPD and psychopathy remained lacking – as indeed was an immutable ontological imaginary in British psychiatry around which agreement might form.

## Something Old, Something New

In the new millennium, the same old questions concerning the ambiguity of antisociality continued to plague psychiatrists. Such issues came to be regarded as especially problematic in light of the increased attention of the psychiatric profession to the management of the “risk” of the individuals and populations they treated and governed – itself a function of new attempts to revise the 1983 Mental Health Act of England and Wales (Pickersgill 2013a, 2013b). Risk thinking and actuarial ways of knowing had “become central” to psychiatry (Rose 1998, 177), and indeed within biomedicine and the health professions more broadly. Such discourses and debates link closely with a larger public and policy emphasis on the regulation of antisocial behaviour (Millie 2007), and a broader shift across various sectors of society towards a concern with “pre-crime” and the management of menacing potentialities (Zedner 2007).

Within psychiatry, talk of “dangerousness” did not revolve solely around adults; discussions of the children “at risk” of developing ASPD were also up for discussion (e.g. Hill 2003), and the idiom of “callous unemotional traits” began to feature within frameworks that emphasized the potential childhood antecedents of psychopathy (and which were linked to particular genetic profiles; see, for instance, Viding et al. 2007). However, the methodological limitations that dominant standards built into research were once more raised; the DSM, for instance, was deemed to “lack specificity” (Hill 2003, s13) – and hence limited the precision of research. In particular, critique was leveled at the DSM-IV-TR (the revised version of the fourth edition of the DSM) and its expansion of the category of ASPD further into the territory “traditionally” occupied by psychopathy (Pickersgill 2012).

The fresh emphasis on risk within psychiatry, and in society more broadly, was supported and furthered by controversial health and criminal justice policy developments which aimed at “Managing Dangerous People with Severe Personality Disorder” (Home Office and the Department of Health 1999).^25^ These proposals – contested by mental health professionals and patients alike – sought to innovate new services aimed at both detaining and treating risky individuals who met the criteria for personality disorder. As Mairead Dolan and Michael Doyle put it in a review article for the BJP, “Risk prediction is once again high on the public, political and clinical agenda” (Dolan and Doyle 2000, 303). Indeed, the management of risk itself became a key target for therapeutic work (e.g. Wong et al. 2007). Noting the different tools available to practitioners to predict risk – unaided clinical risk assessment, actuarial methods, and structured clinical judgment – Dolan and Doyle pointed to the increased use of the PCL-R, which was widely regarded as showing “reasonable predictive validity in determining future violence” (Dolan and Doyle 2000, 304). This diagnostic standard was thus gradually transformed into a tool for the management of risk.

In so doing, the ontology of the disorder it sought to capture was further complicated, since psychopathy could now be considered not just a form of pathological antisociality per se, but also a criterion for estimating an individual's propensity to violence more broadly.^26^ In effect, psychopathy no longer simply contained antisociality but was also contained within it in a complex synecdochical relationship that did nothing to dispel ontological uncertainty. Indeed, the ambiguity of psychopathy is today rendered even more acute, as researchers question not only the cross-cultural generalizability of the PCL-R (Cooke et al. 2005) but even whether antisociality (as measured by criminal behavior) truly comprises a core-feature of the disorder at all (Cooke et al. 2007).

This lack of certainty is maintained alongside a further transformation in the political and social dimensions of antisociality. Today, individuals are not just “at risk” of developing conditions like ASPD, or deemed “risky” to others as a consequence; those living under the label of personality disorder are increasingly viewed as at risk of further complications following the advent of their mental ill-health: “excess mortality” and “high levels of psychiatric morbidity” have been recorded (Davies et al. 2007). Such findings further power the normative impulse within psychiatry to attend to pathological antisociality, and broaden the remit of researchers to clarify the concept and shed light on its causal mechanism. Risks, and ontologies, multiply.

The “neurochemical” motif that Nikolas Rose (2007) has argued to be a major vocabulary through which to articulate and regulate subjectivity in the twenty-first century threaded through certain psychiatric debates on antisociality; the function of serotonin – perhaps the quintessential locus of neurochemical selfhood – was examined, for instance, by Dolan et al. (2001) as a potential biomarker for impulsivity and aggression. Continuing longstanding debates within psychiatry (and elsewhere; Roberts 2006) about the role of hormones in personality, health, and illness, Dolan and colleagues also investigated (and confirmed) the correlation between plasma testosterone levels and aggressive acts. Yet, what is perhaps most strongly evident in recent psychiatric research into the biological aspects of antisociality is the recourse to what Elizabeth Fein (2011) might refer to as a “neurostructural” model of selfhood. Many of the investigations into psychopathy in particular that were published within the BJP – and which resulted in clinical, policy, and public traction – were studies into neurologic structures, rather than the chemicals that flow through them. For instance, in a BJP editorial, prominent psychologist James Blair opened his commentary with the following programmatic statement: “To understand a psychiatric disorder we need to know why the pathology causes the behavioural disturbance, the neural structures implicated in the pathology and the cause of the dysfunction in the neural structures” (Blair 2003, 5).

For Blair, a key neural system implicated in psychopathy is the amygdala – understood to play a key role in the processing of emotion. For IoP psychiatrist Quinton Deeley and colleagues (2006), this hypothesized centrality of the amygdala to psychopathic personality disorder might partly explain their neuroscientific findings that showed a “reduced rather than increased visual cortical response to fearful faces in psychopathy” (Deeley et al. 2006, 538). This is in spite of the fact that these investigators “did not find significant between-group differences in amygdala function” (ibid.). This, it was speculated, could have been due to a “small sample size” (ibid.). Regardless, such comments are indicative of the idea taking shape and gaining credence today in many corners of mental health praxis: namely, that psychopathy is a disorder of empathy and hence related to the amygdala.

But let us not take this growing understanding that psychopathy is related to the amygdala to be a universal consensus, nor that it has rendered concrete the fuzzy nature of the condition; the precise biomarkers articulated by researchers have not lead to a fully biologically-characterized topology of antisociality. In Blair's case, he notes that the “basic causes of the pathology remain unclear” and although the “noradrenergic system could be implicated,” exactly “why this system should be dysfunctional is, again, unknown” (Blair 2003, 6).

Furthermore, while neurologic reflections and research on antisociality continue to be apparent within journals such as the BJP and in prominent meetings and conferences, more commonly, a broadly biopsychosocial narrative is evident in articles, editorials, and correspondence within journals, and, indeed, in practice (e.g., Coid 2003; Duggan et al. 2003; J. Hill 2003; Hodgins 2007; Rutter 2005). Discourse on ASPD and psychopathy can thus be seen as an instantiation of a broader holistic ethic within U.K. psychiatry: whilst neuroscientific and biological perspectives attract significant funding and prestige, advocates of more “patient-centered” approaches also occupy prominent positions (e.g. Bracken and Thomas 2001). Often, psychiatrists attempt to integrate these perspectives, as Fonagy's (2003) “developmental understanding of violence” epitomizes. Indeed, even influential biologically inclined clinician-scientists such as Sir Michael Rutter and Peter McGuffin at the IoP have advocated models of mental disorder that account for the dynamic inter-relations of biology, psychology and the social environment (Rutter and McGuffin 2004). Acting as a discursive resource through which developmental understandings of psychopathology can be fashioned, it is this particular kind of biopsychosocial holism that seems to capture the aetiological understandings of perhaps the majority of researchers and clinicians. With it, an ethic of complexity disunifies psychiatry; research, theory, and therapeutic modalities pluralize; and as a consequence, understandings of the nature of antisociality multiply and thus render the ontology of disorders like psychopathy and ASPD yet more uncertain.

## Discussion

In this paper, I have analyzed the history of British psychiatric discourse on personality disorders associated with antisocial behavior; specifically, psychopathy and ASPD. These are categories that have been relatively neglected by historians and sociologists, but which can have important implications for individuals living under such diagnostic labels. I have attended closely to the diversity of models of these disorders, and noted the enduring uncertainties associated with them – not least of which has been a lack of stable consensus regarding how psychopathy and ASPD develop. In so doing, I have brought into sharp relief the various kinds of biomarkers posited to correlate with or even explain these disorders (e.g. an XYY karyotype, amygdala dysfunction), and situated these within a broader account of the ontological terrain of psychiatry.

Psychopathy has long been a problematic category. First, the causes of the disorder were, and are, uncertain (by which I mean a firmly established and historically consistent aetiological model is lacking): accounts of the condition range widely between biological, psychological, and environmental “factors.” Second, there remains a lack of stable consensus regarding what the term psychopathy properly refers to: exactly who should and should not be considered a psychopath is not always clear. The 1980s saw considerable changes to understandings of antisociality, with two important tools for standardizing pathology developed: the DSM-III and the Hare Psychopathy Checklist. Providing a concrete definition around which personality disorder discourse might revolve, these standards contributed significantly to research and clinical debate. The DSM and PCL were not perfect standards, and debate continued regarding, for instance, their conceptual rigor. However, these concerns were largely set to one side; in processes of “practical uncertainty work” (Pickersgill 2011c; Moreira et al. 2009) researchers and clinicians engaged in therapeutic and investigative practices, whilst at the same time recognizing and lamenting the lack of clarity regarding the pathological image of personality disorder.

The DSM and PCL thus reframed ASPD and psychopathy as legitimate clinical categories and “do-able” research problems (Fujimura 1987); they became the “right tools for the job” (Clarke and Fujimura 1992) of producing rigorous, “objective” knowledge. This was particularly important in light of the epistemological and ontological emphases evident within biomedicine in the late twentieth century. However, the profusion of research investigating antisociality in the 1980s and 1990s appeared to do little to generate a consensus around the ontology of ASPD and psychopathy; developmental accounts continued to be wide-ranging. Such diversity reflected the broad focus of British psychiatry more generally, even as so-called “biological” psychiatry predominated.

Today, the ontologies of ASPD and psychopathy remain unresolved. Some assert that (neuro)scientific research into brain-based biomarkers holds promise in clarifying some of the ambiguities associated with these disorders. However, at present, neuroscientific knowledge appears to be making little difference to practice and neurologic claims may be ignored if they counter clinical goals and assumptions (Pickersgill 2012). As I have sought to show, psychiatry exists in a state of what I refer to as “ontological anarchy” (cf. Feyerabend 2010, 2011), characterized by an ethic of complexity and constituted through a plurality of perspectives on antisociality as a consequence of the wide range of biological, psychological, and sociological “markers” posited to relate in some way to this “condition.”

At the same time, individual psychiatrists have long been what we might call “ontological bricoleurs,” assembling frameworks for understanding antisociality by piecing together different knowledges concerning psyche, soma, and society. Rather than create a consensus about the aetiology of psychopathy and ASPD, biomarker research might, therefore, render these disorders yet more problematic, increasing the intellectual resources from which clinicians might draw to articulate their nature. By consistently failing to fully specify psychopathy, psychiatry comes to be further disunified as new knowledge is produced and circulated – hence widening the scope of the research that may contribute to a more concrete delineation of the category. Whilst collective uncertainty is an important driver of biomedical innovation (Moreira et al. 2009), we can also see how biomarker research into antisociality might be regarded as exemplifying the limits of technoscience in resolving clinical challenges and concerns; it makes “the pathological image more complex rather than clarifying it” (Löwy and Gaudillière 2008, 318).

This analysis, then, also offers up possibilities for thinking about the role of biomarkers more broadly. In particular, it suggests how proposed somatic and psychosocial indicators (such as high levels of theta wave activity, or low serotonergic function) come to play a role in constituting the very pathologies with which they are associated. New research programs, clinical routines, policy guidelines, and ethical regimes evolve together and are energized through discourse and debate on conditions like psychopathy and ASPD and their markers. In this sense, the practices of mental health professionals are, in part, governed through the material-semiotic entity that the (proposed) biomarker “is.” This further complicates attempts to clarify the ontology of psychiatric disorder. Uncertainty endures.

## Footnotes

1 In this paper, I use the term “antisociality” to capture diverse forms of behavior and temperament that can be considered to be in some way antisocial. Later in the analysis I will refer to “pathological antisociality”; by this, I mean forms of antisociality that are deemed by mental health professionals to be representative of some kind of personality (or other psychiatric) disorder, such as psychopathy.

2 In this paper I do not discuss “dissocial personality disorder,” the World Health Organization framing of pathological antisociality, since this term has had minimal purchase within the literature surveyed. A search of the online archives of the *British Journal of Psychiatry* reveals the following instances of the different diagnostic categories: Psychopathy, 654; antisocial personality disorder, 320; dissocial personality disorder, 9.

3 Bynum, Lock, and Porter (1992) describe the role of key professional journals in the production of medical knowledge. In this paper, I focus primarily on one journal – the BJP – in order to define limits to my analysis. An advantage of the BJP as opposed to, for instance, *Psychological Medicine*, is its strongly British focus – especially evident in the editorials and correspondence pages which provide such rich material for the analyst to engage with (however, key articles from *Psychological Medicine* are cited herein, where they have had obvious impacts on UK debate and dialogue). My starting date of 1950 is a convenient locus for shifts in postwar psychiatry, and in particular enables the capturing of debates around antisociality that contributed to the writing of the 1959 Mental Health Act of England and Wales.

4 By “uncertainty,” I mean a lack of stable, working consensus about the ontology of psychopathy/ASPD that can be regarded as widely agreed upon by mental health professionals and researchers.

5 A range of scholars have discussed this point; for example, Baruch and Treacher (1978); Bennett (1991); Busfield (1998); Ewins (1976); Healy (1997); K. Jones (1991); Moncrieff and Crawford (2001); Porter (2002); Tansey (1998). However, the degree to which there is a direct, causal relationship between the new drugs and decreased asylum care is unclear (Rogers and Pilgrim 2001).

6 More generally, postwar British psychiatry largely tended to eschew the insights of psychoanalytic theory (Gelder 1991; E. Jones 2004; Moncrieff and Crawford 2001; Porter 2002). However, as Thomson (2006) has shown, Freudian thought nevertheless had a broad valence within popular culture.

7 The U.S. Minnesota Multiphasic Personality Inventory (the MMPI), which focussed explicitly on the characterization of abnormal personality, was also popular.

8 As Hayward (2001, 625) has pointed out, Grey Walters – a pioneer of EEG – conducted electroencephalogic studies in the 1940s of criminals considered psychopathic.

9 Social psychiatry can thus be thought of as belonging to the tradition of holism within biomedicine that Rosenberg (2007) has characterized as present in various forms throughout the twentieth century.

10 For more on the interactive models of influential figures like Lewis, see Gelder (1991); Hilton (2005); McGuffin and Plomin (2004); Pilgrim (2002); Rutter and McGuffin (2004); Shorter (2005).

11 As noted by Gaylin (1980) and Rainer et al. (1972). For examples, see Bartholemew (1972); Cowen and Mullen (1979); Eysenck and Eysenck (1972); Griffiths (1971); Griffiths et al. (1972); Nielson and Tsuboi (1969).

12 As Temkin (1994) remarks, beliefs in a link between violence and epilepsy are very old.

13 For work on holism in biomedicine more broadly, see Rosenberg (2007).

14 This was not a problem solely restricted to psychopathy (Copeland et al. 1971).

15 Conversely, the authority of the DSM-III was less recognized in countries like Argentina and France where the recourse to biology in psychiatry was less evident (Lakoff 2005; Lloyd 2006).

16 For more detailed accounts of the emergence of ASPD, see Pickersgill 2012; Gurley 2009.

17 However, from the research conducted for this analysis, the extent to which the SCID-II was actually employed within the clinic is unclear. This is a complex empirical question that merits further attention.

18 The term dissocial personality disorder is, anecdotally, regularly used in practice in Scotland, although much public discourse employs the DSM category ASPD (and especially, as noted earlier, the clinical research literature). This raises historical questions about what we mean by, and how we apprehend, the ‘use’ of a diagnostic construct.

19 Journalist Jon Ronson (2011) has also examined the PCL-R.

20 Stone also highlighted the cultural relativity of antisociality, another common complaint about the personality disorders. For a related historical critique of these classifications’ cultural insensitivities, see Ernst (1995).

21 This critique remains current; see, for instance, Pilgrim (2001).

22 This kind of research attracted attention from both lawyers (Andrews 1999; Andrews et al. 2006) and sociologists (Dingwall et al. 2003; Rose 2007).

23 There had, however, been debate about the utility of taking routine EEG readings for antisocial individuals detained in hospitals since at least the 1970s; see, for example, Driver et al. (1974).

24 The lecture, titled “Mindlessness and Brainlessness in Psychiatry” was reprinted in the BJP. This theme was revisited over a decade later in Eisenberg (2000).

25 For sociological commentary, see Manning 2006; Pickersgill 2013b; Pilgrim 2001, 2007; and Rose 2010.

26 For wider discussion on risk in British psychiatry, see Rose (1998, 2010).
